# Association of CVD Candidate Gene Polymorphisms with Ischemic Stroke and Cerebral Hemorrhage in Chinese Individuals

**DOI:** 10.1371/journal.pone.0105516

**Published:** 2014-08-21

**Authors:** Wenjing Ou, Xin Liu, Yue Shen, Jiana Li, Lingbin He, Yuan Yuan, Xuerui Tan, Lisheng Liu, Jingbo Zhao, Xingyu Wang

**Affiliations:** 1 Department of Epidemiology, Public Health School, Harbin Medical University, Heilongjiang, China; 2 Department of Epidemiology, Public Health School, Harbin Medical University, Heilongjiang, China; 3 First Affiliated Hospital, Medical College of Shantou University, Shantou, Guangdong, China; 4 The Laboratory of Human Genetics, Beijing Hypertension League Institute, Beijing, China; Kunming Institute of Zoology, Chinese Academy of Sciences, China

## Abstract

**Background:**

Contribution of cardiovascular disease related genetic risk factors for stroke are not clearly defined. We performed a genetic association study to assess the association of 56 previously characterized gene variants in 34 candidate genes from cardiovascular disease related biological pathways with ischemic stroke and cerebral hemorrhage in a Chinese population.

**Methods:**

There were 1280 stroke patients (1101 with ischemic stroke and 179 with cerebral hemorrhage) and 1380 controls in the study. The genotypes for 56 polymorphisms of 34 candidate genes were determined by the immobilized probe approach and the associations of gene polymorphisms with ischemic stroke and cerebral hemorrhage were performed by logistic regression under an allelic model.

**Results:**

After adjusting for age, sex, BMI and hypertension status by logistic regression analysis, we found that *NPPA* rs5063 was significantly associated with both ischemic stroke (odds ratio [OR] 0.69; 95% confidence interval [CI], 0.52 to 0.90; *P* = 0.006) and cerebral hemorrhage(OR = 0.39; 95%CI, 0.19 to 0.78; *P* = 0.007). In addition, *MTHFR* rs1801133 also was associated with cerebral hemorrhage (OR = 1.48; 95%CI, 1.16 to1.89; *P* = 0.001) but not with ischemic stroke (OR = 1.08; 95%CI, 0.96 to1.22; *P* = 0.210). After false discovery rate (FDR) correction, the association of *NPPA* rs5063 and *MTHFR* rs1801133 with cerebral hemorrhage remained significant.

**Conclusions:**

The *NPPA* rs5063 is associated with reduced risk for cerebral hemorrhage and *MTHFR* rs1801133 is associated with increased risk of cerebral hemorrhage in a Chinese population.

## Introduction

Stroke is one of the leading causes of mortality and disability in the world [1]. In China, about 1.5 to 2 million new strokes occur every year [2], [3], furthermore, there are 58–142 per 100,000 people each year who die of stroke in China [4]. Data from the China Multicenter Collaborative Study of Cardiovascular Epidemiology showed that on average, the proportion of cerebral hemorrhage was one third and the proportion of ischemic stroke was two thirds in Chinese populations [5]. Nowadays, stroke apparently brings enormously economic burden in China [6].

During the past few years, epidemiological studies had confirmed that hypertension, diabetes mellitus, smoking, excessive drinking, and heart diseases acted as conventional risk factors for stroke [7]–[9]. In addition, the role of genetic factors for stroke has been established [10]. To date, many candidate genes have been studied for a potential role in stroke. Such as protein kinase C η (*PRKCH*) [11], *angiotensin receptor like-1* (*AGTRL1*) [12], *methylenetetrahydrofolate reductase* (*MTHFR*) [13], and *guanine nucleotide exchange factor 10* (*ARHGEF10*) [14] were associated with ischemic stroke and *angiotensin-converting enzyme* (*ACE*) [15], *plasminogen activator inhibitor -1*(*SERPINE1*) [15], *apolipoprotein E* (*APOE*) [15] and *coagulation factor V* (*FV*) [15] were associated with cerebral hemorrhage. However, the identified genetic factors explain only a small fraction of the inherited risk of stroke, and the past studies revealed sharing of conventional and genetic risk factors for cardiovascular diseases and strokes. Studies also revealed controversial findings on the association of candidate genes and stroke. It has been reported *MTHFR* increase the risk of ischemic stroke in the Japanese population [13], yet in a Northern India population, Somarajan et al found that *MTHFR* was not associated with ischemic stroke [16]. Thus, there is a need to further study for the association of candidate genes related to stroke in a more defined manner and in large cohorts.

Several physiological pathways, including lipid metabolism, systemic chronic inflammation, coagulation, blood pressure regulation, and cellular adhesion molecules are implicated in the pathophysiology of cardiovascular diseases. Their contributions to stroke were not systematically evaluated. In the present study, we performed a large case-control study in 2660 Chinese individuals, involved in 56 gene polymorphisms of 34 candidate genes from cardiovascular disease to explore these polymorphisms that confer the susceptibility to ischemic stroke and cerebral hemorrhage.

## Materials and Methods

### Study participants

Subjects were recruited from The Stroke Hypertension Investigation in Genetics (SHINING) study, a case-control study carried out by the Beijing Hypertension League Institute between 1997 and 2000 [17]. Study participants were Han ethnicity, enrolled from 6 geographical regions within China (70% study participants came from and near the city of Beijing). All patients had been diagnosed as stroke by brain computed tomography (CT)/MRI. Controls were selected from the same community, and had no prior history of stroke. Controls were matched with cases for sex, age within 3 years, geographic locations, and blood pressure categories (<140/90, ≥140/90 and ≤180/105, >180/105 mmHg) [17]. Stroke patients who had history of myocardial infarction and valvular heart diseases were excluded from the study. Controls who had previous history of stroke or cardiovascular disease were also excluded from the study.

There was a total of 3119 participants were recruited for the SHINING study. We chose only ischemic stroke and cerebral hemorrhage as cases in this study because they constituted majority of stroke patients and the number of patients with other subtypes of stroke, such as subarachnoid hemorrhage, transient ischemic attack (TIA), and with unknown cause was too small to be included in the analysis. A total of 1280 stroke patients, including 1101 ischemic strokes and 179 cerebral hemorrhages, and 1380 controls were included in this study.

Information about demographic factors, lifestyle, and history of disease (such as hypertension) was obtained using structured questionnaires. Hypertension was defined as having current or past antihypertensive medication, or systolic blood pressure ≥140 mmHg, and/or diastolic blood pressure≥90 mmHg [17].

Written informed consent was given by all study participants before participating in the study and the study protocol was approved by ethics committees of the Beijing Hypertension League Institute.

### Genotyping

56 polymorphisms of 34 candidate genes were selected based on the literatures reported in the past, which were combined with trails of cardiovascular disease and lipid metabolism. DNA was extracted from the whole blood with salting out procedure. A PCR-based panel (Roche Molecular Biochemicals, Basel, Switzerland) was used for genotyping and the procedure was described previously [18], [19]. Briefly, firstly, DNA was amplified by PCR with 56 pairs of biotinylated primers in a single tube. Next, each amplified PCR product was hybridized with sequence-specific oligonucleotide probes immobilized on a nylon membrane strip; finally, biotin-based color was detected by a scanner and genotype was analyzed by proprietary Roche Molecular Systems software. To ensure the accuracy of the genotype, genotyping calls were observed by two independent researchers. Genotyping call rate for assessments of all genetic variants was ≥98% in the study.

### Statistical Analysis

Continuous variables expressed as mean ± standard deviation (SD), and were compared between study participants with ischemic stroke or cerebral hemorrhage and controls by Student‘s *t* test. Categorical variables were represented as percentage and were tested by *χ^2^* test. We analyzed departure from Hardy–Weinberg equilibrium by using *χ^2^* test. A minor allele frequency (MAF) <5% would be excluded from the analysis [7].

We estimated the association of genotype with ischemic stroke and cerebral hemorrhage using ORs and 95% CIs, which were calculated by logistic regression under the allelic model. Our analysis concerned two major stroke subtypes, including ischemic stroke and cerebral hemorrhage. For each subtype, cases were compared with the same control group. After then, unadjusted OR (95%CI) and adjusted OR (95%CI) for the candidate genes by logistic model were separately performed. We used the false discovery rate (FDR) to adjust for multiple hypothesis testing [20]. A value of 0.2 [21] for FDR was recommended as significance threshold in some previous candidate gene studies, meaning that one should expect no more than 20% of declared discoveries to be false. Data analyses were applied using SAS statistical software (version 9.2 SAS Institute Inc). P<0.05 indicated statistical significance.

## Results

The characteristics of the 2660 study participants are shown in Table 1. The means of age and BMI were lower (P<0.05) in case group than in control group. SBP was higher (P<0.05) in case group than in control group, whereas DBP was higher (P<0.05) in the cerebral hemorrhage than in control group.

**Table 1:** Characteristics of study participants.

	Stroke patients		Controls
	Ischemic stroke	Cerebral hemorrhage	
No. Of subjects	1101	179	1380
Age, y	59.1±10.7*	58.6±10.5*	60.8±10.6
Sex,% male	60.0	59.8	59.9
BMI, kg/m^2^	24.4±3.0*	23.8±3.1*	25.0±3.3
SBP, mm Hg	145.3±23.2*	147.9±24.5*	143.2±23.9
DBP, mm Hg	86.9±12.9	90.4±13.9*	86.3±13.0
Hypertension,% yes	64.6	71.0	65.2

BMI, body mass index; DBP, diastolic blood pressure; SBP, systolic blood pressure. Age, BMI, DBP and SBP values are mean ± SD. Hypertension indicates systolic blood pressure≥140 mm Hg or diastolic blood pressure≥90 mm Hg (or both), or taking antihypertensive medication.

*P<0.05 vs controls.

The distribution of 56 single nucleotide polymorphisms (SNPs) in each group are shown in Table 2, 20 of 56 SNPs had a MAF<5%. Therefore, these 20 SNPs were excluded and the remaining 36 SNPs were kept for further analysis.

**Table 2:** Distribution of genetic polymorphisms in each group.

Gene	SNP rs No	Minor allele	MAF†			H-W* *P*
			Ischemic stroke	Cerebral hemorrhage	Control	
*LPA* 93C>T	rs1853021	T	0.202	0.223	0.199	0.396
*LPA* 121G>A	rs1800769	A	0.400	0.430	0.422	0.745
*APOB* 71Thr>Ile	rs1367117	T	0.128	0.134	0.126	0.799
*APOC3* (−641)C>A	rs2542052	A	0.457	0.472	0.453	0.649
*APOC3* (−482)C>T	rs2854117	T	0.435	0.455	0.428	0.265
*aAPOC3* (−455)T>C	rs2854116	C	0.435	0.469	0.433	0.512
*APOC3* 1100C>T	rs4520	C	0.428	0.402	0.416	0.271
*APOC3* 3175C>G	rs5128	G	0.294	0.310	0.314	0.642
*APOC3* 3206T>G	rs4225	T	0.194	0.162	0.193	0.172
*APOE* 112Cys>Arg	rs429358	C	0.095	0.089	0.106	0.298
*APOE* 158Arg>Cys	rs7412	T	0.077	0.106	0.083	0.594
*ADRB3* 64Trp>Arg	rs4994	C	0.145	0.164	0.162	0.434
*PPARG* 12Pro>Ala	rs1801282	G	0.068	0.078	0.056	0.835
*LIPC* (−480)C>T	rs1800588	T	0.370	0.379	0.383	0.506
*LPL* 447Ser>Term	rs328	G	0.077	0.081	0.087	0.029
*PON1* 192Gln>Arg	rs662	A	0.379	0.338	0.392	0.897
*PON2* 311Ser>Cys	rs6954345	C	0.189	0.184	0.183	0.539
*LDLR* NcoI +/−	rs5742911	G(−)	0.391	0.391	0.393	0.004
*CETP* 405Ile>Val	rs5882	G	0.472	0.439	0.472	0.379
*LTA* 26Thr>Asn	rs1041981	A	0.418	0.455	0.409	0.266
*MTHFR* 677C>T	rs1801133	C	0.417	0.360	0.442	0.007
*NOS3* (−922)A>G	rs1800779	G	0.095	0.112	0.087	0.403
*NOS3* 298Glu>Asp	rs1799983	T	0.107	0.103	0.110	0.514
*ACE* IVS16 Del>Ins	rs1799752	I	0.360	0.332	0.373	0.384
*AGT* 235Met>Thr	rs699	T	0.198	0.215	0.207	0.418
*NPPA* 664G.>A	rs5063	A	0.044	0.028	0.061	0.073
*ADD1* 460Gly>Trp	rs4961	G	0.477	0.494	0.478	0.884
*SCNN1A* 663Ala>Thr	rs2228576	A	0.483	0.458	0.476	0.613
*GNB3* 825C>T	rs5443	T	0.485	0.489	0.468	0.795
*MMP3* (−1171) Ins>DelA	rs3025058	I	0.159	0.137	0.154	0.315
*F7* (−323) Del>Ins10	rs5742910	I	0.053	0.053	0.050	0.138
*F7* 353Arg>Gln	rs6046	A	0.054	0.050	0.052	0.021
*SERPINE1* (−675)Del>InsG	rs1799768	D	0.442	0.436	0.452	0.971
*SERPINE1* 11053T>G	rs7242	T	0.469	0.489	0.466	0.798
*FGB* (−455)G>A	rs1800790	A	0.201	0.170	0.199	0.925
*ITGA2* 873G>A	rs1062535	A	0.317	0.223	0.199	0.788

*H-W, Hardy–Weinberg equilibrium.

† MAF, minor allele frequency.

The association of SNPs and risk of ischemic stroke and cerebral hemorrhage were listed in Table 3 under the allelic model. The *NPPA* rs5063 was associated with stroke with unadjusted ORs (95%CI; *P* value) of 0.71 (0.55–0.92; 0.009) for ischemic stroke and 0.44 (0.23–0.84; 0.013) for cerebral hemorrhage respectively. After adjustment for age, sex, BMI and hypertension status, ORs of *NPPA* rs5063 (95% CI; *P* value) were 0.69 (0.52–0.90; 0.006) for ischemic stroke and 0.39 (0.19–0.78; 0.007) for cerebral hemorrhage respectively. We applied FDR adjusting for multiple testing, the association of *NPPA* rs5063 with cerebral hemorrhage remained significant with 0.2 as cutoff value (FDR = 0.126) and with ischemic stroke remained borderline significant (FDR = 0.216). *MTHFR* rs1801133 was associated with cerebral hemorrhage. The unadjusted OR (95% CI; *P* value) was 1.41 (1.12–1.77; 0.003), after adjustment for age, sex, BMI and hypertension status, OR (95% CI; *P* value) was 1.48 (1.16–1.89; 0.001) for cerebral hemorrhage. After adjusting for multiple testing, the association of *MTHFR* rs1801133 and cerebral hemorrhage remained significant (FDR = 0.036).

**Table 3:** Association of gene variants and ischemic stroke and cerebral hemorrhage.

Gene	SNP rs No	Ischemic stroke						Cerebral hemorrhage					
		Unadjusted			Adjusted*			Unadjusted			Adjusted*		
		OR(95%CI)	*P*	FDR	OR(95%CI)	*P*	FDR	OR(95%CI)	*P*	FDR	OR(95%CI)	*P*	FDR
*LPA* 93C>T	rs1853021	1.02(0.88–1.17)	0.832	0.951	1.02(0.88–1.19)	0.785	0.961	1.15(0.88–1.50)	0.296	0.732	1.18(0.89–1.58)	0.247	0.588
*LPA* 121G>A	rs1800769	0.91(0.81–1.02)	0.102	0.666	0.90(0.80–1.02)	0.099	0.748	1.03(0.83–1.29)	0.783	0.939	1.03(0.81–1.31)	0.798	0.905
*APOB* 71Thr>Ile	rs1367117	1.02(0.86–1.20)	0.837	0.951	1.01(0.84–1.21)	0.910	0.961	1.07(0.77–1.48)	0.674	0.927	1.08(0.76–1.52)	0.684	0.905
*APOC3* (−641)C>A	rs2542052	1.02(0.91–1.14)	0.741	0.951	1.09(0.96–1.23)	0.169	0.748	1.08(0.87–1.34)	0.496	0.850	1.14(0.90–1.44)	0.278	0.588
*APOC3* (−482)C>T	rs2854117	1.03(0.92–1.15)	0.639	0.921	1.09(0.96–1.23)	0.180	0.748	1.12(0.89–1.39)	0.333	0.732	1.17(0.92–1.48)	0.203	0.562
*APOC3* (−455)T>C	rs2854116	1.01(0.90–1.13)	0.872	0.951	1.07(0.94–1.21)	0.298	0.748	1.16(0.93–1.45)	0.189	0.612	1.21(0.95–1.53)	0.116	0.457
*APOC3* 1100C>T	rs4520	0.95(0.85–1.07)	0.389	0.933	0.97(0.86–1.10)	0.650	0.961	1.06(0.85–1.33)	0.618	0.927	1.07(0.84–1.35)	0.595	0.892
*APOC3* 3175C>G	rs5128	0.90(0.80–1.02)	0.111	0.666	0.94(0.83–1.08)	0.396	0.857	0.98(0.77–1.24)	0.836	0.961	1.01(0.79–1.31)	0.924	0.973
*APOC3* 3206T>G	rs4225	0.99(0.86–1.14)	0.901	0.954	1.03(0.88–1.20)	0.754	0.961	1.23(0.92–1.66)	0.169	0.608	1.23(0.90–1.69)	0.195	0.562
*APOE* 112Cys>Arg	rs429358	0.89(0.74–1.08)	0.241	0.858	0.90(0.74–1.10)	0.310	0.748	0.83(0.57–1.22)	0.346	0.732	0.87(0.58–1.30)	0.494	0.829
*APOE* 158Arg>Cys	rs7412	0.93(0.75–1.14)	0.495	0.951	0.98(0.78–1.23)	0.871	0.961	1.31(0.91–1.89)	0.142	0.568	1.42(0.96–2.09)	0.076	0.456
*ADRB3* 64Trp>Arg	rs4994	0.88(0.75–1.03)	0.104	0.666	0.86(0.72–1.02)	0.073	0.748	1.02(0.76–1.37)	0.882	0.961	1.04(0.76–1.43)	0.805	0.905
*PPARG* 12Pro>Ala	rs1801282	1.22(0.97–1.54)	0.092	0.666	1.16(0.90–1.50)	0.241	0.748	1.42(0.93–2.15)	0.103	0.568	1.45(0.93–2.26)	0.098	0.457
*LIPC* (−480)C>T	rs1800588	0.94(0.84–1.06)	0.331	0.858	0.93(0.82–1.06)	0.258	0.748	0.99(0.78–1.24)	0.908	0.961	0.99(0.78–1.26)	0.951	0.973
*LPL* 447Ser>Term	rs328	0.87(0.71–1.07)	0.198	0.858	0.94(0.75–1.17)	0.549	0.961	0.92(0.62–1.38)	0.685	0.927	1.01(0.66–1.54)	0.973	0.973
*PON1* 192Gln>Arg	rs662	1.06(0.94–1.19)	0.334	0.858	1.05(0.92–1.19)	0.467	0.920	1.26(1.00–1.59)	0.048	0.568	1.32(1.03–1.69)	0.027	0.324
*PON2* 311Ser>Cys	rs6954345	1.03(0.90–1.19)	0.645	0.951	1.10(0.94–1.29)	0.235	0.748	1.01(0.76–1.36)	0.969	0.969	0.96(0.71–1.30)	0.792	0.905
*LDLR* NcoI +/−	rs5742911	0.99(0.88–1.11)	0.870	0.951	1.02(0.90–1.16)	0.757	0.961	0.99(0.79–1.24)	0.938	0.964	0.97(0.76–1.23)	0.804	0.905
*CETP* 405Ile>Val	rs5882	1.00(0.89–1.12)	1.000	1.000	1.01(0.89–1.14)	0.935	0.961	0.87(0.70–1.09)	0.234	0.648	0.83(0.66–1.05)	0.127	0.457
*LTA* 26Thr>Asn	rs1041981	1.04(0.93–1.16)	0.521	0.951	1.09(0.97–1.24)	0.159	0.748	1.21(0.97–1.51)	0.092	0.568	1.28(1.01–1.62)	0.038	0.342
*MTHFR* 677C>T	rs1801133	1.09(0.99–1.24)	0.074	0.666	1.08(0.96–1.22)	0.210	0.748	1.41(1.12–1.77)	0.003	0.108	1.48(1.16–1.89)	0.001	0.036
*NOS3* (−922)A>G	rs1800779	1.10(0.91–1.34)	0.327	0.858	1.01(0.82–1.25)	0.929	0.961	1.32(0.92–1.87)	0.130	0.568	1.19(0.81–1.75)	0.366	0.693
*NOS3* 298Glu>Asp	rs1799983	0.97(0.81–1.17)	0.766	0.951	0.90(0.74–1.09)	0.285	0.748	0.93(0.65–1.34)	0.711	0.927	0.90(0.62–1.32)	0.595	0.892
*ACE* IVS16 Del>Ins	rs1799752	0.94(0.84–1.06)	0.325	0.858	0.93(0.82–1.06)	0.290	0.748	0.84(0.61–1.05)	0.128	0.568	0.82(0.64–1.05)	0.107	0.457
*AGT* 235Met>Thr	rs699	1.06(0.92–1.22)	0.436	0.951	1.06(0.91–1.23)	0.486	0.920	0.95(0.73–1.25)	0.721	0.927	0.96(0.72–1.28)	0.778	0.905
*NPPA* 664G.>A	rs5063	0.71(0.55–0.92)	0.009	0.324	0.69(0.52–0.90)	0.006	0.216	0.44(0.23–0.84)	0.013	0.234	0.39(0.19–0.78)	0.007	0.126
*ADD1* 460Gly>Trp	rs4961	1.01(0.89–1.12)	0.931	0.957	1.01(0.89–1.14)	0.890	0.961	0.90(0.72–1.12)	0.327	0.732	0.92(0.73–1.17)	0.507	0.829
*SCNN1A* 663Ala>Thr	rs2228576	1.03(0.92–1.15)	0.631	0.951	1.01(0.90–1.15)	0.820	0.961	0.93(0.74–1.16)	0.525	0.859	0.88(0.69–1.12)	0.300	0.600
*GNB3* 825C>T	rs5443	1.07(0.96–1.20)	0.228	0.858	1.07(0.94–1.20)	0.312	0.748	1.08(0.87–1.35)	0.463	0.850	1.04(0.82–1.31)	0.773	0.905
*MMP3* (−1171) Ins>DelA	rs3025058	0.96(0.83–1.12)	0.630	0.951	1.01(0.85–1.19)	0.928	0.961	1.15(0.84–1.58)	0.385	0.770	1.21(0.86–1.70)	0.264	0.588
*F7* (−323) Del>Ins10	rs5742910	1.07(0.83–1.38)	0.581	0.951	1.02(0.77–1.34)	0.907	0.961	1.07(0.66–1.76)	0.781	0.939	0.89(0.52–1.52)	0.655	0.905
*F7* 353Arg>Gln	rs6046	1.05(0.82–1.34)	0.721	0.951	0.98(0.75–1.29)	0.902	0.961	0.96(0.58–1.59)	0.878	0.961	0.72(0.41–1.26)	0.247	0.588
*SERPINE1*(−675)Del>InsG	rs1799768	1.04(0.93–1.17)	0.471	0.951	1.05(0.93–1.19)	0.405	0.857	1.07(0.86–1.33)	0.558	0.873	1.10(0.87–1.39)	0.440	0.792
*SERPINE1* 11053T>G	rs3918226	0.99(0.88–1.10)	0.801	0.951	0.99(0.88–1.12)	0.907	0.961	0.83(0.67–1.04)	0.139	0.568	0.81(0.64–1.02)	0.070	0.456
*FGB* (−455)G>A	rs7242	1.01(0.88–1.17)	0.858	0.951	1.00(0.86–1.16)	0.973	0.973	0.83(0.62–1.11)	0.204	0.612	0.79(0.58–1.08)	0.140	0.458
*ITGA2* 873G>A	rs1800790	0.93(0.82–1.04)	0.206	0.858	0.96(0.85–1.10)	0.572	0.961	0.92(0.73–1.17)	0.489	0.850	1.03(0.80–1.32)	0.843	0.919

FDR  =  false discovery rate.

*Adjusted for age, sex, body mass index and hypertension status in allelic model of inheritance.

We also tested the interaction of *NPPA* rs5063 and *MTHFR* rs1801133 and hypertension in control group, and found no interaction between variants and hypertension status. We further individually tested the association of *NPPA* rs5063 and *MTHFR* rs1801133 with ischemic stroke and cerebral hemorrhage stratified with hypertension status (shown in Table 4). In the hypertension group, after adjustment for age, sex and BMI, the *NPPA* rs5063 was associated with ischemic stroke and cerebral hemorrhage. The ORs (95% CI; *P* value) were 0.70 (0.51–0.97; 0.034) for ischemic stroke and 0.37 (0.13–0.86; 0.021) for cerebral hemorrhage. The *MTHFR* rs1801133 was associated with cerebral hemorrhage. The OR (95% CI; *P* value) was 1.38(1.03–1.84; 0.030) for cerebral hemorrhage. In the non- hypertension group, the *NPPA* rs5063 was not associated with ischemic stroke and cerebral hemorrhage. The ORs (95% CI; *P* value) were 0.69(0.42–1.12; 0.134) for ischemic stroke and 0.47(0.14–1.57; 0.219) for cerebral hemorrhage. The *MTHFR* rs1801133 was associated with cerebral hemorrhage. The OR (95% CI; *P* value) was 1.75(1.10–2.78; 0.018) for cerebral hemorrhage.

**Table 4:** Association of polymorphism rs5063 and rs1801133 with ischemic stroke and cerebral hemorrhage based on hypertension-stratified population.

Stratified group	Controls	Ischemic stroke			Cerebral hemorrhage		
		Subjects	OR(95%CI)*	*P* *	Subjects	OR(95%CI)*	*P* *
Non-hypertension							
rs5063(NPPA)	480	390	0.69(0.42–1.12)	0.134	52	0.47(0.14–1.57)	0.219
rs1801133(MTHFR)	480	390	1.09(0.88–1.36)	0.403	52	1.75(1.10–2.78)	0.018
Hypertension							
rs5063(NPPA)	900	711	0.70(0.51–0.97)	0.034	127	0.37(0.13–0.86)	0.021
rs1801133(MTHFR)	900	711	1.07(0.92–1.25)	0.359	127	1.38(1.03–1.84)	0.030

Genetic model = allelic model; reference allele = G (rs5063); reference allele = C (rs1801133).

*Adjusted for age, sex and body mass index in allelic model of inheritance.

We further analyzed the interaction between *NPPA* rs5063 and *MTHFR* rs1801133 with ischemic stroke and cerebral hemorrhage. After adjustment for age, sex, BMI and hypertension status, the interaction between *NPPA* rs5063 and *MTHFR* rs1801133 with ischemic stroke and cerebral hemorrhage was not statistically significant. The ORs (95% CI; *P* value) were 0.87 (0.62–1.22; 0.410) for ischemic stroke and 0.70 (0.32–1.55; 0.381) for cerebral hemorrhage (data not shown).

## Discussion

In the present study, we examined the relationship of 36 CVD related candidate gene variants with ischemic stroke and cerebral hemorrhage. After adjusting for age, sex, BMI and hypertension status, we found that the *NPPA* rs5063 was significantly associated with reduced risk for ischemic stroke and cerebral hemorrhage in SHINING cohort. This association of *NPPA* rs5063 with cerebral hemorrhage remained significant under the allelic model after adjusting for multiple testing by FDR whereas the association of *NPPA* rs5063 with ischemic stroke remained borderline significant (FDR = 0.216).

In the present study, *NPPA* rs5063 was associated with cerebral hemorrhage and marginally associated with ischemic stroke. It is inconsistent concerning the association between *NPPA* rs5063 and stroke. Rubattu et al [22] reported that in a matched, case-control study, *NPPA* rs5063 polymorphism was associated with the occurrence of stroke (348 strokes and 348 controls) under additive (OR, 1.9; 95% CI, 1.16 to 3.12; P = 0.01) and dominant model (OR, 2.0; 95% CI, 1.17 to 3.39; P = 0.01). Later, a small case-control study was reported which did not find significant difference in the presence of *NPPA* rs5063 gene variants between ischemic stroke and control participants [23]. This inconsistency on the association between *NPPA* rs5063 and stroke might be the results of sample size, different study designs or different ethnic groups. In particular, the A allele frequencies of *NPPA* rs5063 observed in the present study was 0.061 in the Han Chinese Population, whereas in the White population the A allele frequency is approximately 0.034 [22]. Therefore, further investigation with a greater sample size is required to evaluate the association between NPPA rs5063 and ischemic stroke. To our knowledge, the previous studies have explored the association of *NPPA* rs5063 with total stroke or ischemic stroke cases. The studies about the association of *NPPA* rs5063 and cerebral hemorrhage were rarely conducted probably due to the insufficient cases in the study population. Thus, the association of *NPPA* rs5063 with cerebral hemorrhage needs to be further verified by in diverse populations with a larger sample size.

The physiological function of *NPPA* variant and the biological pathways of its involvement in stroke are at present unknown. However, the source of *NPPA* and this variant and the biological role of this variant have been already suggested. The *NPPA* (natriuretic peptide precursor A) gene is located on chromosome 1p36, encodes the precursor from which atrial natriuretic peptide (*ANP*) [24] is derived [25]. The mutation of *NPPA* rs5063 appears in the exon1, which is responsible for a valine-to-methionine substitution in the proANP peptide. Recently, this mutation in the NPPA has been found to be associated with higher circulating levels of ANP in salt-sensitive essential hypertension [26] and in familial atrial fibrillation [27]. *ANP* also exerts powerful natriuretic, diuretic and other beneficial effects [10], [28]–[30]. Although we did not measure the circulating levels of ANP as the function of *NPPA* rs5063, the biological role of this variant may have some effect on the biological pathways of its involvement in stroke.

Out of the remaining 36 SNPs, we found that T allele of *MTHFR* rs1801133 was associated with increased risk of cerebral hemorrhage under the allelic model after adjustment for age, sex, BMI and hypertension status (OR = 1.48; 95% CI, 1.16–1.89). For ischemic stroke, no association with *MTHFR* rs1801133 was found (OR = 1.08; 95%CI, 0.96–1.22).

The mutation of *MTHFR* rs1801133 is a 677C-to-T transition, which causes an alanine-to-valine substitution in the *MTHFR* protein. *MTHFR* rs1801133 leads to a reduction in a thermolabile enzyme activity and subsequent elevation of plasma homocysteine [31]. It is generally accepted that elevated homocysteine concentrations may induce atherosclerosis and cause endothelial dysfunction [32], [33]. Atherosclerosis is a common risk factor for ischemic stroke and cerebral hemorrhage [34], [35]. The association between *MTHFR* rs1801133 and cerebral hemorrhage was consistent with the previous studies [36], [37], that suggested that the *MTHFR* rs1801133 was associated with increased risk of cerebral hemorrhage, and the T allele may be an important risk factor for cerebral hemorrhage. However, Somarajan et al found that *MTHFR* rs1801133 was neither associated with cerebral hemorrhage nor ischemic stroke in a Northern India population [16]. In our study, the *MTHFR* rs1801133 was not associated with ischemic stroke. Cronin et al, reported that in the cumulative meta-analysis, among 14870 subjects, the T allele of *MTHFR* rs1801133 genetic polymorphism was associated with increased risk of ischemic stroke(T allele pooled OR 1.17, 95%CI 1.09 to 1.26) [38]. There are several reasons may account for the inconsistency between these studies. First, there are racial-ethnic differences in distribution of the polymorphism [39]. The T allele frequencies of *MTHFR* rs1801133 observed in the present study was 0.442 in the Chinese Han population, the mutation tends to be less prevalent in the Northern India population (frequency of the T allele 0.17). Secondly, unique design of current study by matching cases and controls with blood pressure may overly expose risk factors that are difficult to hunt by conventional case control studies. Ultimately, apart from genetic factors, there are different levels of vitamin B family and folic acid intake in the different regions and populations, which may cause inconsistent results. Although we did not measure the concentration of either homocysteine or vitamin B family and folic acid or derivatives, we speculate that the different levels of vitamin and folate intake do exist in different populations which may impact the results.

Apart from MAF, Hardy-Weinberg equilibrium analysis, we conducted a LD analysis by PLINK software, and found linkage between APOC3 (−641) C> A (rs2542052) and APOC3 (−482) C> T (rs2854117); APOC3 (−641) C> A (rs2542052) and APOC3 (−455) T> C (rs2854116); APOC3 (−482) C> T (rs2854117) and APOC3 (−455) T> C (rs2854116) on chromosome 11. LD also exists between F7 (−323) Del> Ins10 (rs5742910) and F7 353Arg> Gln (rs6046) on 13 chromosome. We further conducted association analysis for all haplotypes with ischemic and hemorrhagic stroke, and we found no statistically significance association (p>0.05).

Hypertension is a main risk factor for ischemic stroke and cerebral hemorrhage [40]. Due to our matching criteria, cases and controls were matched by their blood pressure categories. The strategy was initially designed to increase the chance of finding genes predisposing to ischemic stroke and cerebral hemorrhage independent of blood pressure. In addition, it has been noted that in a large-scale prospective study, the A allele of *NPPA* rs5063 has provided a protective effect for blood pressure progression in 48 months and incident hypertension for the entire follow-up[41]. Qian ea al, reported that in a meta-analysis that MTHFR rs1801133 was significantly associated with hypertension among both the European and East Asian adult population [42]. In the present study, cases and controls were matched with blood pressure categories. To further rule out the influence of *NPPA* rs5063 and *MTHFR* rs1801133 on blood pressure and subsequently on ischemic stroke and cerebral hemorrhage, we tested the interaction of *NPPA* rs5063 and *MTHFR* rs1801133 with hypertension status in control population, and we did not find any interaction with hypertension (data not shown). We further individually tested the association of *NPPA* rs5063 and *MTHFR* rs1801133 with ischemic stroke and cerebral hemorrhage in the hypertension and non-hypertension groups and found that *NPPA* rs5063 was associated with both ischemic stroke and cerebral hemorrhage in the hypertension group, In non -hypertension group, the association between *NPPA* rs5063 and ischemic stroke and cerebral hemorrhage did not reach significance but the effect size and directions were the same as in hypertension group. *MTHFR* rs1801133 was associated with cerebral hemorrhage in both hypertension group and non-hypertension group. Therefore, we concluded that *NPPA* rs5063 and *MTHFR* rs1801133 were associated with cerebral hemorrhage and *NPPA* rs5063 was marginally associated with ischemic stroke and were not directly associated with hypertension. These results were derived from stratified cohorts, therefore, the sample size, alone with other factors may play a role in the significant association. Studies with greater sample size and in other population are needed to ascertain the associations.

Limitations of our study also should be discussed. (i) Subjects recruited were stroke survivors from (SHINING study) [17], which introduced survival bias and impacted the stroke subtypes. Thus, the present study must be interpreted within the context of its limitations. (ii) Valid stratification can diminish the effects of confounding factors. However, reducing the sample size, at the same time, which made the boundary effect more difficult to be detected. (iii) In the present study, the sample size in the hemorrhagic stroke is relatively small, although there are positive associated detected after adjusting for FDR, the results should be interpreted cautiously. Future studies are needed to explore in detail for the important issue.

## Conclusions

Our study showed that the *NPPA* rs5063 was significantly associated with cerebral hemorrhage, and the *MTHFR* rs1801133 was associated with increased risk of cerebral hemorrhage, but not with ischemic stroke in a Chinese population. We also found that *NPPA* rs5063 was associated with cerebral hemorrhage and ischemic stroke and *MTHFR* rs1801133 was associated with cerebral hemorrhage in the hypertension group and *MTHFR* rs1801133 was associated with cerebral hemorrhage in the non-hypertension group and were not directly associated with hypertension. It is necessary for future large scale studies to further explain the *NPPA* and *MTHFR* variants and stroke subtypes.
